# Psychometric evaluation of the Coronary Revascularisation Outcome Questionnaire (CROQ) in Norwegian patients admitted to elective coronary angiography and possible percutaneous coronary intervention

**DOI:** 10.1186/s12955-022-01927-9

**Published:** 2022-02-05

**Authors:** Ingvild Nordnes Myrbakk, Oddgeir Friborg, Anne Høye, Terje Steigen, Svein Bergvik

**Affiliations:** 1 Department of Psychology, Faculty of Health Sciences, The Arctic University of Norway, Tromsø, Norway; 2 Department of Clinical Medicine, Faculty of Health Sciences, The Arctic University of Norway, Tromsø, Norway; 3 Division of Mental Health and Substance Abuse, University Hospital of North Norway, Tromsø, Norway; 4 Clinical Cardiovascular Research Group, UiT The Arctic University of Norway, Tromsø, Norway; 5 Department of Cardiology, University Hospital of North Norway, Tromsø, Norway; 6 UiT Norges Arktiske Universitet, Institutt for Psykologi, 9037 Tromsø, Norway

**Keywords:** Coronary revascularisation outcome questionnaire, Health-related quality of life, Coronary heart disease, Percutaneous coronary intervention, Psychometric properties, Patient reported outcome measures

## Abstract

**Background:**

The Coronary Revascularisation Outcome Questionnaire (CROQ) measures health-related quality of life and outcome of invasive revascularization procedures such as percutaneous coronary intervention (PCI) or coronary artery bypass grafting (CABG). The CROQ has not been properly validated in Norwegian patient populations. The aim of this study was to examine the psychometric properties of the Norwegian CROQ in patients admitted to elective coronary angiographic assessment and receiving PCI. Moreover, to examine its discriminative ability to detect disease severity and effects of invasive coronary treatment.

**Methods:**

The patients (*N* = 280, *M*_age_ = 66.9, *SD*_age_ = 8.91) completed the CROQ, prior to an elective coronary angiography and at one year follow-up. Analyses included internal consistency, floor and ceiling effects, and confirmatory and exploratory factor analyses of the CROQ. Convergent validity was evaluated by comparing CROQ scores with the RAND-12 measure. Sensitivity to treatment was examined by comparing pre-post effect size differences between the PCI treatment and non-treatment group.

**Results:**

Significant stenosis qualifying for a PCI was detected in 121 (35.1%) patients. The model fit of the original CROQ factor model was inadequate in the PCI group. All but one of the CROQ items demonstrated ceiling effects. The CROQ failed to discriminate between patients’ disease severity prior to the coronary angiography, or improvement in those receiving versus not receiving PCI.

**Conclusion:**

The present study of the Norwegian version of the CROQ identified serious problems with the factor structure, ceiling effects, and lack of sensitivity for disease severity and effects of invasive treatment. Currently, one cannot recommend the use of CROQ in clinical practice.

## Introduction

Coronary angiography (CA) combined with intracoronary physiological measurement, is considered the definite diagnostic procedure to identify coronary heart disease [1]. Waiting for a CA assessment can be stressful for the patients, and may increase their vulnerability for ill psychological health [2, 3]. Further, physicians tend to underrate the symptom burden of the patients [4, 5]. Routine assessment with a patient reported outcome measure (PROM) could improve physicians’ understanding of how the disease affects the patients’ daily life, and contribute to quality improvement and clinical research. This is also reflected in the European Society of Cardiology (ESC) Guidelines for the diagnosis and management of chronic coronary syndromes, which recommends to include quality of life assessment in the diagnostic process [1].

Several disease specific PROMs have been developed for coronary heart disease (CHD) populations [6–8]. To our knowledge, Coronary Revascularisation Outcome Questionnaire (CROQ) is the only PROM specifically designed to measure health-related quality of life (HRQoL) and presumed sensitive to the effect of invasive revascularization treatment procedures such as PCI or coronary artery bypass graft (CABG) surgery. CROQ has pre- and post-vascularisation versions, and assesses patients’ functioning across four domains; cardiac symptoms, physical functioning, psychosocial functioning and cognitive functioning [7].

The original English version of the CROQ has demonstrated some acceptable psychometric properties [7, 9]. However, the factor structure has not been evaluated by confirmatory factor analyses. The CROQ has been translated to several languages, and exploratory factor analyses of some of these, including the Norwegian post-vascularization version, have reported mixed findings with regard to the factor structure [10–12]. The Norwegian pre-vascularization version used prior to the PCI also lacks validation. Despite no floor effects, ceiling effects appear abundant in the Norwegian post-procedure version, ranging between 22 to 45% in three of the four subscales [11]. This is unfortunate as it lowers the sensitivity for detecting changes following interventions or between patients that are differently affected by their coronary status [13, 14]. Nevertheless, pre-assessment to post-intervention change score effect sizes range between 0.19 to 1.06 (on subscale levels) in patients receiving PCI or CABG [9]. However, sensitivity to treatment effects of the PCI/CABG has not been examined by comparing data with patients not receiving any invasive procedure following an elective coronary angiography.

The present study examined the psychometric quality, factor structure and construct validity of the CROQ, which to our knowledge, has not been previously done on a large sample of Norwegian patients before and after an elective angiographic assessment procedure. We expected that the CROQ would discriminate between disease severity prior to the angiographic procedure, and detect larger changes in HRQoL in patients receiving PCI treatment as compared to patients with coronary symptoms but without a significant stenosis level according to the coronary angiography assessment.

## Methods

### Sample

A total of 391 patients (48.9% of the invited) consented to participate. In all, 48 were excluded; 13 due to an acute hospitalization during the waiting period, ten for not attending the angiographic procedure, six for completing the questionnaire after the angiographic procedure, two for incomplete contact information, and 17 for incomplete questionnaire data with missing more than 50% of the items on a subscale. Further, 63 patients were not included because they had stenosis > 50%, but did not receive PCI. Hence, the final sample at pre-assessment included 280 patients. Following the angiographic assessment, 121 (35.1%) received PCI and 159 patients (46.8%) had a stenosis degree < 49% (regarded as non-significant stenosis).

At post-angiography, 12 were excluded due to incomplete questionnaire data. The final sample consisted of 232 participants; with 104 patients who had received PCI and 128 patients from the non- significant stenosis group.

### Procedure

Patients scheduled for an elective coronary angiographic assessment at the Heart and Lung Clinic at the University Hospital of North Norway (UNN) in Tromsø between October 2017 and August 2018 were invited to participate. The invitation letter and the first set of questionnaires were enclosed the summon from the hospital. Consenting patients were instructed to complete the questionnaire prior to the angiographic procedure and return it by mail in a prepaid envelope. Approximately one year after the procedure, the patients received a follow up questionnaire by mail with a pre- paid return envelope. Patients not responding within 3–4 weeks received a reminder. Participants returning questionnaires were compensated with 2 lottery tickets (~ 6 USD).

### Angiographic procedure

Cardiologists with ten years of experience or more performed the angiographic procedures. Degree of stenosis was based on visual examination and intracoronary physiological measurements (e.g. fractional flow reserve) when considered appropriate. Patients with stenosis < 49% obstruction on all coronary arteries were included in the non- significant stenosis group.

### Measurement instruments

Demographic data collected at pre-assessment included age, gender, education (primary school, high school, university < 4 years, university > 4 years) and cohabitation (living with a spouse yes/no).

### Coronary revascularisation outcome questionnaire

Coronary Revascularisation Outcome Questionnaire (CROQ) is a self-administered and disease specific outcome questionnaire developed to measure health outcomes and quality of life in patients receiving PCI or CABG [7]. The CROQ consist of 32 items covering the four domains; symptoms (seven items), physical functioning (eight items), psychosocial functioning (14 items) and cognitive functioning (three items). Items use a 3–6 points Likert scaled response format, where higher scores indicate better functioning. Items of each subscale are summarised and converted into a 0–100 scale. Two of the eight items measuring physical functioning are identical to the physical component in SF 12/RAND 12, which was included in this study. To reduce the patient burden and avoid repeated items, the physical scale was presented in the RAND12 and responses copied to CROQ. Missing data were less than 4,2% for all of the items and replaced using the multiple imputation algorithm based on the expected maximization procedure in PRELIS 2 [15].

### RAND-12

The RAND-12 is a 12 item generic health status measure of mental and physical health. Items are scored on a Likert scale ranging from 2 to 6 with higher scores reflecting better functioning [16]. It was adapted from the RAND/SF-36 full version (36 items) by including 12 items from the eight scales of the SF-36. The items scores are converted to summary measures based on scoring algorithms allowing the mental scale (MCS 12) and physical scale (PCS 12) to be correlated [17]. The RAND-12 has shown acceptable test retest reliability from r. 76 to. 89, and acceptable Cronbach alpha ranging from 0.77 to 0.80 [16, 18].

### Registry data

The following data from the hospital records was retrieved from the Norwegian Registry for Invasive Cardiology (NORIC); degree of stenosis classified from 1 to 6 according to percentage of stenosis on different arterial segments (1 = 0–29%, 2 = 30–49%, 3 = 50–69%, 4 = 70–89%, 5 = 90–99%, 6 = 100%), previous PCI (yes/no), previous CABG (yes/no), previous heart attack (yes/no) and type of performed procedure (angiography, PCI or CABG).

### Statistical analysis

Descriptive statistics, correlation and group comparison analyses were carried out using IBM SPSS Statistics version 26. Cronbach’s alpha was used to evaluate the internal consistency of the subscale scores (values > 0.7 is generally considered acceptable). Convergent validity was examined by correlation analysis. The floor and ceiling effects were calculated as the percentage frequency of the lowest or highest possible score on the items, and considered present if more than 15% [13].

Effect sizes were calculated as standardized mean differences between patients receiving PCI and patients with non-significant stenosis, $Cohe{n}^{^{\prime}}s d=\frac{{M}_{1}-{M}_{2}}{pooled SD}$,or as change scores within each group as $Cohen`s d=\frac{M1-M1}{SD difference}$. To examine if the CROQ change scores differed significantly between the PCI and the non-PCI groups, we specified a mixed model regression with a compound correlation matrix and estimated three fixed coefficients: time (change from pre to post), group (PCI vs. non-significant stenosis), and time*group that examines if the level of change was significantly different between the groups. We repeated this analysis, adding gender, age and cohabitation for adjustment purposes.

### Confirmatory factor analysis

Confirmatory factor analyses were conducted in Mplus 8.4 [19]. Eight of the items belonging to the factor physical functioning used a 1–3 Likert scale response format, and were treated as categorical of an ordinal nature; hence, we used the weighted least squares mean and variance (WLSMV) estimator with robust errors adjusting for non-normal item distributions [20]. Our sample size (N = 121) is in the lower end, but given three categories, 17 indicators with moderately high standardized factor loadings < . 7, we consider the bias ratio as acceptably low [21].

We report relative fit in terms of the Tucker-Lewis Index (TLI) and the Comparative Fit Index (CFI), the model misspecification index root-mean-square error of approximation (RMSEA) [22] and the weighted root-mean-square residual (WRMR). The recommendations for cut-off values with categorical data and small sample sizes[23] are RMSEA < 0.07 as good fit (and < 0.08 as adequate fit), CFI > 0.90 and TLI > 0.94. A clear criterion for the WRMR as an index of the residual correlations is lacking.

In addition to examining the model fit of the original CROQ model (M1), as published by Schroter and Lamping (2004) in the PCI sample, we compared it with the following competing models: a single primary factor accounting for all item covariances (M2) and a second-order factor to the M1 model (named M3).

### Exploratory factor analysis

The EFA was conducted in Mplus8.4 using the estimator WLSMV, as eight items had three response categories [24]. Factor loadings were geomin rotated in order to allow correlations between the factors [25]. Number of factors to extract was determined on the basis of a scree plot [26].

## Results

### Respondent characteristics

The mean age of the 121 patients in the PCI group were 68.2 years (78.5% males), and the majority had a spouse (83.9%; see Table 1). In the non-significant stenosis group, which represented the comparison group, the mean age was 66.0 years (54.1% males) and the majority had a spouse (73.3%).

**Table 1:** Characteristic of the respondents in the PCI group and the non-significant stenosis group at pre-assessment

	Patients receiving PCI (n = 121)	Non-significant stenosis group (n = 159)
Mean age (SD) years	68.2 (8.0)*	66.0 (9.5)*
Male (%)	78.5***	54.1***
Cohabitation (%)	83.9*^a^	73.3*^b^
Highest education (%)	_c_	_d_
Primary school	32.8	41.1
High school	41.2	29.7
University	26.1	29.1
Previous PCI (%)	37.2	27
Previous CABG (%)	17.4***	0***
Previous heart attack (%)	19.0	16.4

*p < .05, ***p < .001. SD: standard deviation

^a^2.5% missing

^b^5.7% missing

^c^0.6% missing

^d^1.7% missing

At post-angiography, the PCI group consisted of 104 patients (*M*-age = 67.3, *SD*-age 7.7 and 78.8% males), whereas the non- significant stenosis group consisted of 128 patients (*M*-age = 67.3; *SD*-age = 9.0 and 53.1% males).

### Reliability of the CROQ subscales: internal consistency

The internal consistency of the CROQ subscale scores at pre-assessment and post- angiography for the patients receiving PCI are presented in Table 2. The Cronbach`s alphas for the entire scale score (32 items) were 0.93 and 0.94, respectively, and ranged between 0.77 and 0.93 across all subscale scores at both measurement occasions.

**Table 2:** Descriptive, test score reliability and sensitivity data of the CROQ subscale scores at pre- and post-angiography for patients undergoing PCI

	Pre-assessment PCI group (n = 121)			Post-angiography PCI group (n = 104)		
CROQ scores (range 0–100)	Score (0–100) M (SD)	Floor/ceiling effects %	Cronbach`s alpha	Score (0–100) M (SD)	Floor/ceiling effects %	Cronbach`s alpha
Symptoms	71.0 (20.2)	3.5/36.0	.84	88.9 (13.4)	0.6/67.9	.85
Physical functioning	74.2 (23.9)	10.5/59.0	.89	85.6 (18.7)	4.6/75.6	.86
Psychosocial functioning	72.9 (17.3)	1.8/35.7	.93	85.1 (13.7)	0.4/57.3	.92
Cognitive functioning	83.9 (17.3)	0.3/50.2	.83	89.9 (12.1)	0/62.2	.77

*M* average score, *SD* standard deviation

### Floor and ceiling effect of the CROQ

There were minor floor effects at pre-assessment or post-angiography on CROQ subscale levels. The ceiling effects were more pronounced at both pre- and post-assessment literally across all items (31 of 32 items at pre-assessment) as it ranged between 19% and 86.8% (see Table 2). At post-angiography, all items had ceiling effects ranging between 42.3 and 90.4%.

### Discriminatory function at pre-assessment

None of the CROQ subscales discriminated significantly between patients designated for PCI treatment and patients with non-significant stenosis at pre-assessment. The mean CROQ subscale scores in the PCI group were even scarcely higher (better) as compared to the non-significant stenosis group (see Table 3).

**Table 3:** Differences in pre-assessment CROQ scores between patients undergoing PCI and patients in the non-significant stenosis group

CROQ scores (range 0–100)	PCI group (n = 121) Mean (SD)	Non-significant stenosis group (n = 159) Mean (SD)	*d*
Symptoms	70.99 (20.23)	70.57 (17.13)	0.02
Physical functioning	74.23 (23.93)	72.21 (24.09)	0.08
Psychosocial functioning	72.85 (17.68)	69.61 (18.74)	0.18
Cognitive functioning	83.86 (17.34)	80.08 (18.81)	0.21

*SD* standard deviation, *d* = Cohen’s d

### Construct validity: sensitivity to treatment effects following PCI

Table 4 presents effect sizes for the CROQ subscale change scores. Both groups (PCI and the non-significant stenosis group) improved substantially on all CROQ subscales from pre-assessment to post-angiography with largest improvements in coronary symptoms and psychosocial functioning. However, these improvements were not significantly different between the two patient groups, except for the physical functioning scale improving more in the PCI group compared with the non-significant stenosis group (unadjusted *p* = 0.024; adjusted *p* = 0.017). The associated effect size improvement in the PCI group was half a standard deviation (Cohen’s *d* = 0.50) whereas the non-significant stenosis group improved approx. one-third of a standard deviation (*d* = 0.31), which is a minor difference. This difference would turn non-significant if adjusting for multiple significance testing.

**Table 4:** Baseline to post-test change score differences in CROQ scores between the PCI and the non-PCI patient groups

	Patients receiving PCI *n* = 104			Non-significant stenosis group *n* = 128			
CROQ Scores (range 0–100)	Baseline *M* (SD)	Post *M* (SD)	*r *_*95%CI*_ *d *_*95%CI*_	Baseline *M* (SD)	Post *M* (SD)	*r *_*95%CI*_ *d *_*95%CI*_	diff^1^_*95%CI*_ diff^2^_*95%CI*_
Symptoms	71.5 (20.1)	88.9 (13.4)	.44 _.22–.62_ .94 _.71–1.17_	70.9 (17.2)	86.8 (12.5)	.46 _.30–.61_ 1.00 _.79–1.21_	1.5 _–2.7 | 5.9_ 2.0 _–2.3 | 6.4_
Physical functioning	75.2 (23.4)	85.6 (18.7)	.53 _.36–.68_ .50 _.29–.70_	73.0 (24.4)	78.2 (23.0)	.76 _.66–.84_ .31 _.14–.49_	5.5* _0.7 | 10.3_ 6.0* _1.1 | 10.9_
Psychosocial functioning	73.4 (17.5)	85.1 (13.7)	.56 _.43–.67_ .77 _.55–.99_	70.2 (18.9)	81.8 (16.5)	.72 _.63–.80_ .88 _.67–1.08_	0.1 –_3.5 | 3.7_ 0.03 –_3.6 | 3.7_
Cognitive functioning	84.9 (16.2)	89.9 (12.1)	.60 _.41–.75_ .38 _.18–.58_	80.1 (19.0)	86.3 (15.8)	.53 _.35–.69_ .36 _.18–.54_	–0.7 _–4.5 | 3.0_ –0.9* _–4.9 | 3.0_

*M* = mean, *SD* = standard deviation, *r* = pre-post stability coefficient (Pearson’s r), *d* = Cohen’s d standardized pre-post mean difference (effect size), _*95%CI*_ = 95% confidence interval

diff^1^ = Significance test for change score differences between the two groups, diff^2^ = same as diff^1^, plus adjustment for gender, age and cohabitation. * *p*-value between .01 and .05

### Confirmatory factor analysis (CFA)

The original four-factor model of the CROQ as proposed by the authors [7] was tested using a CFA on the pre version of the CROQ. According to Table 5, the model discrepancy index (RMSEA) was mediocre, whereas the incremental fit indices (CFI and TLI) were particularly poor. The standardized factor loadings ranged from 0.422 to 0.950 (all p`s < 0.001), thus including items with low loadings and poorer discriminative ability. As expected, the original four-factor CROQ model demonstrated better fit than a simple one-factor model, thus confirming multi-dimensionality. The fit of the second-order model was slightly worse than the original four-factor model, which was expected given its more parsimonious structure by excluding the estimation of all factor correlations (Table 5). However, since model fit was not substantively poorer, a general score rather than four factor scores may be preferred for parsimonious reasons without sacrificing substantive information.

**Table 5:** Model fit indices of the CROQ at pre-assessment in the PCI group (*n* = 121)

	χ^2^	df	RMSEA	90% CI	CFI	TLI	WRMR
Original structure	764.3***	458	.074	.065–.083	.787	.769	.993
One factor model	1088.0***	464	.105	.097–.114	.566	.536	1.387
Second order	808.5***	460	.079	.070–.088	.758	.739	1.063

χ^2^: Satorra–Bentler scaled chi-square, df: Degrees of freedom; RMSEA: root-mean-square error of approximation; CI: Confidence Interval; CFI: Comparative Fit Index; TLI: Tucker-Lewis Index; WRMR: Weighted root-mean-square residual

### Exploratory factor analysis

Given the mediocre CFA results of the a-priori hypothesized model, we performed an exploratory factor analysis in order to examine what the potential factor structure might be. The EFA extracted six factors with eigenvalues > 1 (12.57, 4.31, 2.77, 1.43, 1.26 and 1.05, respectively). As the drop in eigenvalues leveled markedly off at the fourth factor (scree plot criterium), and the last two factors accounted for few items (three or less), we extracted four factors in line with the authors of the CROQ (*R*^2^ = 0.659). The model fit indices for this sample-optimized factor model were more acceptable (χ^2^_df=374_ = 508.91, p < 0.001; RMSEA = 0.055; CFI = 0.906; NNFI = 0.876).

As is evident in Table 6, the EFA-based loadings revealed several items with significant cross loadings (correlating with several factors, thus being ambiguous), misplaced items (loading on unintended factor), or items with low loadings. The physical functioning and the symptom scales had both two items with the highest loading on unintended factors, whereas the three cognitive items correlated most strongly with the psychosocial functioning factor score. In conclusion, this EFA-based factor structure deviated markedly from the original four-factor structure.

**Table 6:** Exploratory factor-analysis based loadings with oblique geomin rotation at pre-assessment in the PCI group (*n* = 121)

CROQ items	Factor 1	Factor 2	Factor 3	Factor 4
**Symptoms**				
Chest pain	.762*			
Chest discomfort	.697*			
Shortness of breath		.305*		
Angina radiates	.623*			
Palpitations			.378*	
Frequency of use of nitroglycerin	.576*			
Troubles with heart condition	.716*			
**Physical functioning**				
Moderate activities	.315*	.495*		.449*
Lifting and carrying		.474*		.508*
Climbing several flight of stairs		.944*		
Climbing one flight of stairs		.944*		
Bending and kneeling		.598*		.366*
Walking about 1 km		.789*		
Walking about 100 M		.783*		
Bathing or dressing		.351*		.729*
**Psychososial functioning**				
Overprotective environment	.342*			
Feeling like a burden			.542*	
Restriction of social activities			.494*	.329*
Worried about going too far from home			.638*	
Worried about your heart condition			.650*	
Worried about overdoing			.586*	− .333*
Worried about sudden attack			.496*	− .614*
Frightened by pain			.553*	.486*
Uncertain about future			.828*	− .320*
Depressed		− .464*	.973*	
Frustrated/impatient		− .308*	.889*	
Enjoyment of life	.323*		.744*	
Difficult to keep a positive outlook			.791*	
Difficult to plan ahead			.725*	
**Cognitive functioning**				
Difficulty reasoning and solving problems			.845*	
Forgetting things	− .389*		.570*	
Difficulty concentrating			.732*	

Factor loadings < .30 are omitted. * *p* < .05

### Convergent validity

The correlations between the subscales of the CROQ and the RAND 12 are presented in Table 7.

**Table 7:** Spearman rho coefficients between the CROQ subscales and the physical scale (PCS12) and mental scale (MCS12) of the RAND 12 (*n* = 110, PCI group)

CROQ	PCS12	MCS12
Physical functioning	.80 *	.40 *
Psychosocial functioning	.59 *	.63 *
Symptoms	.62 *	.39 *
Cognitive functioning	.34 *	.33 *

*p < .001

As expected, the physical CROQ and RAND-12 subscale scores correlated strongly, as did the CROQ psychosocial and the RAND-12 mental scale. The remaining CROQ subscales correlated moderately to strongly with the two RAND-12 subscales.

## Discussion

The Norwegian version of the Coronary Revascularisation Outcome Questionnaire was examined in a clinical context on patients awaiting elective coronary angiography; hence, being unaware of their disease status and any need for coronary intervention. The CROQ, as devised by the authors [7], measures four subscales related to coronary symptoms and functions. The results of the present study provide mixed support for the psychometric properties and validity of the CROQ. On a positive note, the CROQ subscales showed moderate to strong correlations with a comparable health-related scale (RAND-12), which indicates satisfactorily convergent validity of the CROQ. Similar findings were also observed by Schroter and Lamping in their evaluation of the original CROQ [7], which also indicates that aspects of health-related quality of life issues underpin both of these measures.

On the negative side, the confirmatory factor analysis of the a-priori factor structure by Schroter and Lamping [7] was inadequate, and not amenable for post-hoc modeling aimed at identifying a revised and better fitting model. We thus conducted an exploratory factor analysis (EFA) to reveal the best fitting factor structure in this sample. Although the EFA reproduced three of the original four factors (the cognitive subscale failing as a unique factor), it revealed some of the problems with the CROQ: several items with low loadings, being misplaced by loading on unintended factors, or correlating highly with multiple factors (high cross-loadings) causing ambiguity in the interpretation. The cognitive items worked particularly poor, and need substantial revision or to be removed. In sum, this raises concerns about the factor structure of the CROQ and the subscales should be used with caution due to their ambiguous multidimensional nature. These findings indicate a need for a substantial revision of the scale items before further use of the CROQ-N in clinical or research contexts. Similar problems such as problematic cross-loadings [10–12] have been noted by others, and small factor loadings was also noted by the author of the original psychometric evaluation of the CROQ [7].

The current study revealed substantial ceiling effects on both subscale and item levels. The ceiling effects we observed were relatively higher than those reported by the original validation study by Schroter et. al. (2017) [9]. One explanation for these differences may be the patient groups included in the studies. Their sample included patients with a known heart disease (i.e., the PROMs pilot project) put on a waiting list for coronary revascularization, whereas our sample included patients admitted to elective coronary angiography with an unknown disease status.

The CROQ showed significant pre to post improvements on all subscales in both groups, which was expected for the PCI group but not for the non-PCI group. This indicates that the CROQ may be sensitive to other non-PCI changes, such as the role of additions or changes in medications, increased knowledge about the functioning of their heart following the angiography procedure, or changes in personal care following the diagnostic attention. It is noteworthy that three of the four CROQ subscales were insensitive to the PCI intervention, except for the physical function subscale that detected a change score improvement that was significantly larger among patients receiving PCI as compared to patients with non-significant stenosis. The CROQ is intended to detect treatment effects following a PCI/CABG intervention; hence, one might expect that the CROQ would be more sensitive to detecting changes in the PCI group compared to the group of patients with non-significant stenosis. We consider this as an indication of poor construct validity. The high ceiling effects, as noted above, may partly explain this lack of sensitivity [13, 14]. In addition, the CROQ failed to discriminate between levels of coronary disease severity prior to the angiographic assessment. The CROQ therefore ostensibly measures more generic aspects of coronary-related symptoms and functions, rather than specific treatment-related outcome effects related to invasive coronary treatment, as the name of the scale suggests.

In conclusion, although the reliability of the CROQ scores are adequate, the construct validity stands out as quite poor. We do not expect that replacing the current analyses with modern test theory approaches (i.e., Item Response Theory) will remedy the psychometric properties sufficiently [27] to overcome the rather poor construct validity. We therefore recommend a substantial revision of the CROQ measure that is based on current knowledge of symptoms or function losses that are prominent before and after coronary interventions as PCI or CABG. This should be done before resuming use in patient or research contexts.

## Limitations

One limitation in our study is that we only included information from hospital records at pre-assessment, and have no information of any further treatment provided by the hospital, the General Practitioner or others. Thus, we cannot rule out the possibility that some patients may have received a PCI/CABG or other medical treatments between pre-assessment and post-angiography that may have affected their self -reported health status. Also, we did not include disease specific measures when testing for convergent validity, nor measures expected to have a negative correlation (discriminative validity).

Further, this study is within the lower limits of the sample size requirements to conduct a CFA with WLSMV estimation. There is a risk that the estimated standard errors of factor loadings in the CFA with WLSMV estimation is underestimated with low sample sizes (< 300), and this leads to risk for Type 1 error according to model fit indices [21]. However, we believe these potential problems have minimal impact upon the overall conclusions, as the results from the EFA supported the concerns about the construct validity.

## Conclusions

The present study provides mixed support for applying the CROQ measure in coronary patient populations receiving PCI. We noted problems with the factor structure, and lack of sensitivity for disease severity and lack of responsiveness on most of the scales. The CROQ-N needs a substantial revision and currently we cannot recommend the use of the questionnaire in clinical practice.

## Data Availability

Not applicable due to patient confidentiality.

## Abbreviations

CA: Coronary angiography
CABG: Coronary artery bypass grafting
CFA: Confirmatory factor analysis
CFI: Comparative fit index
CHD: Coronary heart disease
CI: Confidence Interval
CROQ: Coronary Revascularisation Outcome Questionnaire
DF: Degrees of freedom
EFA: Exploratory factor analysis
ES: Effect sizes
ESC: European Society of Cardiology
HRQoL: Health-related quality of life
IRT: Item response theory
M: Mean
MCS12: Mental scale
NORIC: Norwegian Registry of Invasive Cardiology
PCI: Percutaneous coronary intervention
PCS12: Physical scale
PROM: Patient reported outcome measure
RMSEA: Root-mean-square error of approximation
SD: Standard deviation
TLI: Tucker-Lewis Index
WLSMV: Weighted least squares mean and variance
WRMR: Weighted root-mean-square residual
χ^2^: Satorra –Bentler scaled chi-square
